# Gender-specific associations between atherogenic index of plasma and the presence and severity of acute coronary syndrome in very young adults: a hospital-based observational study

**DOI:** 10.1186/s12944-019-1043-2

**Published:** 2019-04-13

**Authors:** Gaojun Cai, Wei Liu, Sai Lv, Xu Wang, Yonghe Guo, Zhenxian Yan, Yu Du, Yujie Zhou

**Affiliations:** 1 Department of Cardiology, 12th ward, Beijing Anzhen Hospital, Capital Medical University, Beijing Institute of Heart Lung and Blood Vessel Disease, Beijing Key Laboratory of Precision Medicine of Coronary Atherosclerotic Disease, Clinical center for coronary heart disease, Capital Medical University, Beijing, 100029 China; 2 Department of Cardiology, Wujin Hospital affiliated with Jiangsu University, Changzhou, 213017 Jiangsu China; 3 Emergency & Critical Care Center, Beijing Anzhen Hospital, Capital Medical University, Beijing, 100029 China

**Keywords:** Acute coronary syndrome, Lipid, Atherogenic index of plasma, Young

## Abstract

**Objective:**

The value of atherogenic index of plasma (AIP) as a predictive biomarker for coronary artery disease (CAD) remains controversial. In addition, whether AIP is associated with the risk of acute coronary syndrome (ACS) in very young adults has not been well established.

**Methods:**

We consecutively collected very young adults (≤35 years of age) undergoing coronary angiography (CAG) at Anzhen Hospital, between January 2008 and December 2017. Total of 1, 478 very young participants, including 1, 059 ACS patients and 419 non-CAD subjects, were enrolled in the present study.

**Results:**

Very young patients with ACS had higher AIP level compared with non-CAD participants (0.35 ± 0.30 vs 0.21 ± 0.33, *P* < 0.001). According to Gensini Score (GS) and number of lesion vessel, patients were divided into four groups, respectively. With the elevated GS score and number of lesion vessels, the AIP level increased gradually (*P*_for trend_ all< 0.05). Multivariate logistic regression analyses suggested that AIP remained to be independently associated with the presence of ACS and was superior to traditional lipid profiles (for AIP, OR = 2.930, 95% CI = 1.855–4.627, *P* < 0.001; for total cholesterol, OR = 1.152, 95% CI = 1.048–1.266, *P* = 0.003; for triglyceride, OR = 1.078, 95% CI = 0.991–1.172, *P* = 0.079; for low-density lipoprotein cholesterol, OR = 1.046, 95% CI = 1.015–1.078, *P* < 0.001), after adjustment for other traditional confounders. Moreover, the prevalence of ACS, acute myocardial infarction, unstable angina pectoris and the value of GS were also elevated as AIP quartiles increased (*P*_for trend_ < 0.001). Subgroup analysis based on gender revealed that AIP was only independently associated with the ACS risk in male.

**Conclusions:**

AIP was independently associated with the presence and severity of ACS in very young patients in a gender-dependent manner, which might be superior to traditional lipid profiles.

**Electronic supplementary material:**

The online version of this article (10.1186/s12944-019-1043-2) contains supplementary material, which is available to authorized users.

## Introduction

Coronary artery disease (CAD) remains the leading cause of morbidity and mortality worldwide [1]. With the development of modernization and changes in lifestyle, including increased consumption of meat and reduced physical exercise, the incidence of CAD was gradually increased in recent years in China [2]. According to 2017 annual report, there are about 11 million CAD patients in China, which has been the important public health problem [3]. Acute coronary syndrome (ACS), including acute myocardial infarction (AMI) and unstable angina pectoris (UAP) is the most severe type in CAD. The results of the China PEACE-Retrospective Acute Myocardial Infarction Study showed that, from 2001 to 2011, the national rates of hospital admission for STEMI increased greatly (from 3.5 per 100,000 people in 2001, to 15.4 per 100,000 people in 2011) [4].

Dyslipidemia in traditional lipid profiles, including total cholesterol (TC), low-density lipoprotein cholesterol (LDL-C), triglyceride (TG) and high-density lipoprotein cholesterol (HDL-C) has been identified as the major risk factor for ACS. Many studies demonstrated that effectively decreasing LDL-C could significantly reduce the cardiovascular events. However, some patients with statin treatment remain having the elevated risk of cardiovascular events, that is residual risk [5].

Atherogenic index of plasma (AIP), expressed as the logistical transformation of the mole ratio of TG to HDL-C, was a significant predictor of AS and better than traditional pro-atherogenic lipid profiles [6]. Epidemiological studies suggested that AIP was significantly associated with obesity, essential hypertension (EH), diabetic mellitus (DM) and other risk factors for CAD [7–9]. In recent years, AIP was identified the superior predictor of CAD and the cardiovascular events [10, 11]. In a hospital-based observational study conducted in participants undergoing CAG, AIP was the most strong lipid parameter associated with CAD, with an odds ratio of 1.660 for an increase of 1-SD, after adjusting for age, gender, smoking, EH and DM [10]. Besides, studies suggested that AIP might be a better predictor for mortality risk among an older adult population, compared with individual cholesterol risk factors [12]. In a 10-year follow-up study, AIP was positively associated with the risk of all-cause death in elderly women with EH [11].

However, despite these associations, the value of AIP as a predictive biomarker for CAD remains controversial [13]. In addition, young patients with CAD exhibit a different risk factor profile from older ones [14]. Whether AIP is associated with the risk of ACS in very young patients has not been well established. In light of these considerations, we conducted the hospital-based observational study to investigate the relationship between AIP and the risk of ACS in very young adults (≤ 35 years age) and hypothesized its predictive value to very young ACS was superior to traditional lipid profiles.

## Methods

### Participants

In this single-center observational study, we consecutively collected very young participants (≤35 years of age) undergoing coronary angiography (CAG) at Anzhen Hospital, between January 2008 and December 2017.

The exclusion criteria were the following: 1) age < 18 years old; 2) participants with incomplete lipid profiles data; 3) repeated hospitalization. Participants with severe renal insufficiency, nephrotic syndrome, myocarditis, infectious endocarditis, multiple arteritis, Kawasaki disease, or having history of percutanious coronary intervention were also excluded.

**Fig. 1:**
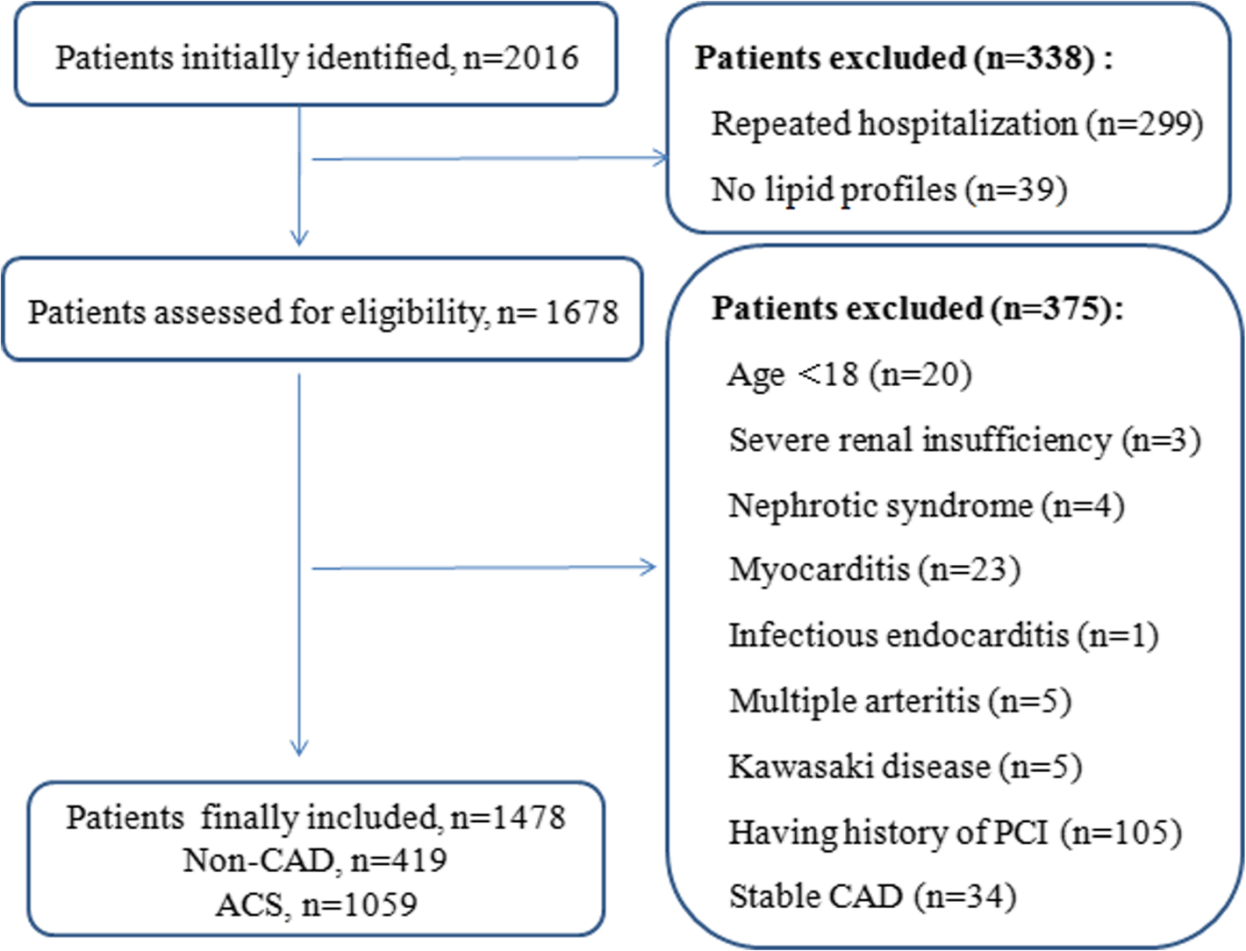
Flow chart illustrating the process of participant enrolled in the study

According to the exclusion criteria, total of 1, 478 consecutively very young participants, including 1, 059 ACS patients and 419 non-CAD subjects, were enrolled in the present study. The flowchart outlining the study was shown in Fig. 1.

This study was complied with the Declaration of Helsinki and approved by the Institutional Ethics Committee of Beijing Anzhen Hospital. Written informed consent was not obtained from the participants, because of the data retrospectively obtained from electronic medical records.

### Biochemical parameter analysis

Fasting venous blood was drawn from all participants. Data of clinical and demographic characteristics including age, gender, height, weight, vital signs, smoking and drinking status, history of EH and DM, were collected from electronic medical records. Laboratory parameters including TC, LDL-C, HDL-C, TG, total protein (TP), pre-albumin (PALB), blood urea nitrogen (BUN), creatinine (CR), fasting blood glucose (FBG) and glycated albumin (GA) were analyzed by automated biochemical analyzer. AIP was calculated as logarithmic transformation of the ratio of TG to HDL-C [log_10_ (TG/HDL-C)].

### Diagnostic criteria

ACS was diagnosed according to European Society of Cardiology guidelines in 2015 [15]. CAG was performed on all participants and assessed by two experienced cardiologists. The severity of coronary lesion was evaluated by Gensini score (GS) and number of stenotic coronary arteries [16]. Based on the value of GS, patients were classified into four quartiles. Besides, the number of lesion coronary artery was calculated by counting the major coronary vessel stenosis ≥50%, which including the left main, left anterior descending, left circumflex, right coronary artery, and main branches (diameter of vessel is more than 2.0 mm). When the stenosis in left main comes to the criteria, the number of lesion vessel was defined as two-vessel, whether or not coexists left anterior descending and/or left circumflex lesions. Patients were then classified into single-vessel, two-vessel, and three-vessel disease groups. Moreover, patients with ACS were divided into AMI and UAP groups.

The diagnostic criteria of EH and DM were described in our previously study [17]. Briefly, EH was defined as repeated systolic pressure ≥ 140 mmHg and/or diastolic pressure ≥ 90 mmHg at least twice, or previously diagnosed EH. DM was defined as FGB ≥7.0 mmol/l and/or random glucose level ≥ 11.1 mmol/l, or previously diagnosed DM. Smoker and drinker were defined as regular cigarette smoking and alcohol intake, respectively.

### Statistic analysis

Statistical Package for the Social Sciences (SPSS Inc., Chicago, IL, USA) 17.0 was used for statistical analysis. Continuous variables were presented as the mean ± standard deviation (SD) and were compared using an independent samples *t* test or one-way analysis of variance (ANOVA) if the data were normally distributed. Otherwise, the data were presented as median and interquartile range (IQR) and were compared using Mann-Whitney U test. The normality of data was evaluated by Kolmogorov-Smirnov test. Categorical variables were expressed as frequencies and percentages and were compared using a Chi-square test. The correlations between AIP and body mass index (BMI) and other lipid profiles were explored by using Pearson analysis. Multivariate logistic regression analyses, expressed as odds ratio (OR) with 95% confidence interval (95% CI), were performed to assess the association of AIP with the risk of ACS, adjusted for age, sex smoker, EH, DM (Model 1) and plus BMI (Model 2). Trend analysis was also used to detect the change trend of variables among quartiles. A value of *P* < 0.05 in a two-sided test was considered statistical significantly.

## Results

### The baseline characteristics of involved participants

Table 1 lists the baseline characteristics of involved participants. It is not surprising that the very young patients with ACS were more likely to be male (94.9% vs 83.83%, *P* < 0.001). The prevalence of smoker, EH, DM and lipid-lowing treatment was significantly higher in very young ACS patients than in non-CAD subjects. Moreover, ACS patients had higher levels of WBC, RBC, PLT, FBG and GA, and lower levels of TP, PALB and BUN compared with non-CAD subjects. Whereas, there were no difference in age, vital signs, the prevalence of drinker and past history of dyslipideamia between two groups. As expected, the concentrations of TC, TG and LDL-C were higher in ACS group than in non-CAD group. On the contrary, HLD-C level was significantly lower in ACS patients than that in non-CAD subjects.

**Table 1:** Baseline characteristics of involved participants

Characteristics	Missing data n (%)	Total (*n* = 1, 478)	Non-CAD (*n* = 419)	ACS (*n* = 1, 059)	*P*
Demographic data					
Age, years	0 (0.00)	33 (30–35)	33 (30–35)	33 (30–35)	0.270
Male [n(%)]	0 (0.00)	1, 373 (92.90)	368 (87.83)	1005 (94.90)	**< 0.001**
Height, cm	161 (10.89)	172.64 ± 6.79	172.68 ± 7.41	172.62 ± 6.51	0.875
Weight, kg	161 (10.89)	82.95 ± 15.32	80.22 ± 17.05	84.12 ± 14.37	**< 0.001**
BMI, kg/m^2^	161 (10.89)	27.73 ± 4.42	26.74 ± 4.84	28.15 ± 4.15	**< 0.001**
Vital signs					
SBP, mmHg	1 (0.10)	124.56 ± 16.05	125.12 ± 14.48	124.34 ± 16.64	0.399
DBP, mmHg	1 (0.10)	77.96 ± 12.37	77.95 ± 10.97	77.97 ± 12.89	0.977
HR, bpm	1 (0.10)	75.74 ± 12.28	75.38 ± 12.55	75.88 ± 12.17	0.480
Life styles					
Smoker, n(%)	0 (0.00)	984 (66.58)	232 (55.37)	752 (71.01)	**< 0.001**
Drinker, n(%)	0 (0.00)	358 (26.05)	107 (25.54)	251 (23.70)	0.459
Past history					
EH, n(%)	0 (0.00)	491 (33.22)	102 (24.34)	389 (36.73)	**< 0.001**
DM, n(%)	0 (0.00)	133 (9.00)	22 (5.25)	111 (10.48)	**0.001**
Dyslipidaemia, n(%)	0 (0.00)	152 (10.28)	47 (11.22)	105 (9.92)	0.449
Prior lipid-lowing treatment, n(%)	0 (0.00)	236 (15.97)	21 (5.01)	215 (20.30)	**< 0.001**
Laboratory parameters					
WBC, ×10^9^/L	27 (1.83)	8.29 ± 3.07	7.13 ± 1.88	8.70 ± 3.29	**< 0.001**
RBC, × 10^12^/L	17 (1.15)	4.99 ± 0.45	4.98 ± 0.51	5.00 ± 0.42	0.453
HGB, g/L	17 (1.15)	152.32 ± 14.54	152.03 ± 16.42	152.43 ± 13.78	0.629
PLT, ×10^9^/L	17 (1.15)	239.64 ± 61.74	224.70 ± 54.48	245.56 ± 63.46	**< 0.001**
TP, g/L	16 (1.08)	69.65 ± 5.60	70.51 ± 5.55	69.31 ± 5.58	**< 0.001**
PALB, g/L	27 (1.83)	0.28 ± 0.06	0.29 ± 0.06	0.27 ± 0.06	**< 0.001**
BUN, g/L	4 (0.27)	4.723 ± 1.59	5.07 ± 1.53	4.59 ± 1.59	**< 0.001**
CR, mmol/L	4 (0.27)	76.99 ± 16.41	77.42 ± 16.98	76.82 ± 16.18	0.532
FBG, mmol/L	4 (0.27)	5.76 ± 1.76	5.34 ± 1.21	5.93 ± 1.91	**< 0.001**
GA, %	558 (37.75)	13.33 ± 3.41	12.77 ± 1.77	13.53 ± 3.79	**0.003**
HbA1C, %	564 (38.16)	6.10 ± 4.38	6.18 ± 8.79	6.08 ± 1.45	0.788
Lipid profiles	0 (0.00)				
TC, mmol/L		4.58 ± 1.35	4.36 ± 1.07	4.67 ± 1.44	**< 0.001**
TG, mmol/L		1.82 (1.26–2.78)	1.58 (1.07–2.40)	1.95 (1.33–2.89)	**< 0.001**
HDL-C, mmol/L		0.94 ± 0.22	1.03 ± 0.25	0.91 ± 0.21	**< 0.001**
LDL-C, mmol/L		2.89 ± 1.13	2.68 ± 0.85	2.97 ± 1.21	**< 0.001**
AIP		0.31 ± 0.32	0.21 ± 0.33	0.35 ± 0.30	**< 0.001**
CAG characteristics	0 (0.00)				
One-vessel, n(%)				519 (35.12)	
Two-vessel, n(%)				244 (16.51)	
Three-vessel, n(%)				192 (12.99)	
GS				32 (14–62.75)	

*CAD* coronary artery disease, *ACS* acute coronary syndrome, *BMI* body mass index, *SBP* systolic blood pressure, *DBP* diastolic blood pressure, *HR* heart rate, *DM* diabetes mellitus, *EH* essential hypertension, *WBC* white blood cell, *RBC* red blood cell, *HGB* hemoglobin, *PLT* platelet, *TP* total protein, *PALB* pre-albumin, *BUN* blood urea nitrogen, *CR* creatinine, *FBG* fasting blood glucose, *GA* glycated albumin, *TC* total cholesterol, *TG* triglyceride, *HDL-C* high-density lipoprotein cholesterol, *LDL-C* low-density lipoprotein cholesterol, *AIP* atherogenic index of plasma, *CAG* coronary angiography, *GS* Gensini score

Bold values indicate statistical significance

### Correlation analysis of AIP with other variables

As shown in Additional file 1: Table S1, in whole population, AIP was significantly positively associated with age, BMI, GA, PALB, TC, TG, LDL-C, and negatively associated with HDL-C (*P* all < 0.05).

### Relation of AIP level and the presence and severity of ACS

As shown in Table 1, AIP value was significantly higher in very young ACS group than in non-CAD group (0.21 ± 0.33 vs 0.35 ± 0.30, *P* < 0.001). According to quartiles of GS, patients were divided into four groups (quartile 1: ≤12; quartile 2: 12–32; quartile 3: 32–64; quartile 4: > 64). Additional file 1: Table S2 lists the clinical characteristics of ACS patients according to GS quartiles. We found that there was a significantly elevated trend of AIP as GS increased (*P*_for trend_ = 0.01). Furthermore, the correlation of AIP and number of lesion vessel was investigated. In 104 ACS patients, none of the stenosis of lesion vessels in each patient comes to 50%. So, patients were classified into one-vessel (*n* = 519), two-vessel (*n* = 244) and three-vessel (*n* = 192) groups. Similarly, an elevated trend of AIP was observed as the increase of number of lesion vessels (*P*_for trend_ < 0.001) (Additional file 1: Table S3).

In order to assess the association of AIP with the presence and severity of ACS in very young patients, participants were further divided into four groups based on AIP quartiles (quartile 1: < 0.117; quartile 2: 0.117–0.312; quartile 3: 0.312–0.505; quartile 4: > 0.505). As shown in Table 2, the prevalence of male, EH, DM and the value of BMI, TC, TG and LDL-C were increased with the elevated AIP quartiles (*P*_for trend_ < 0.001). However, HDL-C level gradually decreased (*P*_for trend_ < 0.001). It is not surprising that the prevalence of ACS, AMI, UAP and the value of GS were also elevated as AIP quartiles increased (*P*_for trend_ < 0.001).

**Table 2:** Clinical characteristics according to AIP quartiles among all participants

Characteristics	Q1 (< 0.117)	Q2 (0.117–0.312)	Q3 (0.312–0.505)	Q4 (> 0.505)	*P*
Clinical Characteristics					
Age, years	32 (29–34)	32 (30–35)	32 (31–35)	32 (31–35)	**<0.001**
Male, n(%)	299 (81.03)	352 (95.14)	360 (97.30)	362 (98.10)	**<0.001**
BMI, kg/m^2^	25.62 ± 4.73	27.75 ± 4.41	28.72 ± 4.18	28.89 ± 3.44	**<0.001**
Smoker, n(%)	176 (47.70)	253 (68.38)	279 (75.41)	296 (80.22)	**<0.001**
EH, n(%)	101 (27.37)	123 (33.24)	138 (37.30)	149 (40.38)	**<0.001**
DM, n(%)	13 (3.52)	28 (7.57)	42 (11.35)	59 (15.99)	**<0.001**
Laboratory parameters					
TC, mmol/L	4.20 ± 1.26	4.42 ± 1.36	4.59 ± 1.15	5.11 ± 1.46	**<0.001**
TG, mmol/L	0.99 (0.75–1.18)	1.52 (1.32–1.77)	2.18 (1.87–2.47)	3.77 (3.06–4.99)	**<0.001**
HDL-C, mmol/L	1.14 ± 0.25	0.95 ± 0.17	0.83 ± 0.17	0.82 ± 0.17	**<0.001**
LDL-C, mmol/L	2.65 ± 1.17	2.93 ± 1.24	3.02 ± 0.99	2.94 ± 1.06	**<0.001**
CAG characteristics					
ACS, n (%)	210 (56.91)	271 (73.24)	282 (76.22)	296 (80.22)	**<0.001**
AMI, n (%)	106 (28.73)	154 (41.62)	169 (45.68)	177 (47.97)	**<0.001**
UAP, n (%)	104 (28.18)	117 (31.62)	113 (30.54)	119 (32.25)	**<0.001**
GS	5 (0–32)	16 (0–44.75)	20 (0–48.5)	25 (3–56)	**<0.001**

*ACS* acute coronary syndrome, *AMI* acute myocardial infarction, *UAP* unstable angina pectoris, *Q* quartile, *BMI* body mass index, *DM* diabetes mellitus, *EH* essential hypertension, *TC* total cholesterol, *TG* triglyceride, *HDL-C* high-density lipoprotein cholesterol, *LDL-C* low-density lipoprotein cholesterol, *AIP* atherogenic index of plasma, *CAG* coronary angiography, *GS* Gensini score

Bold values indicate statistical significance

**Fig. 2:**
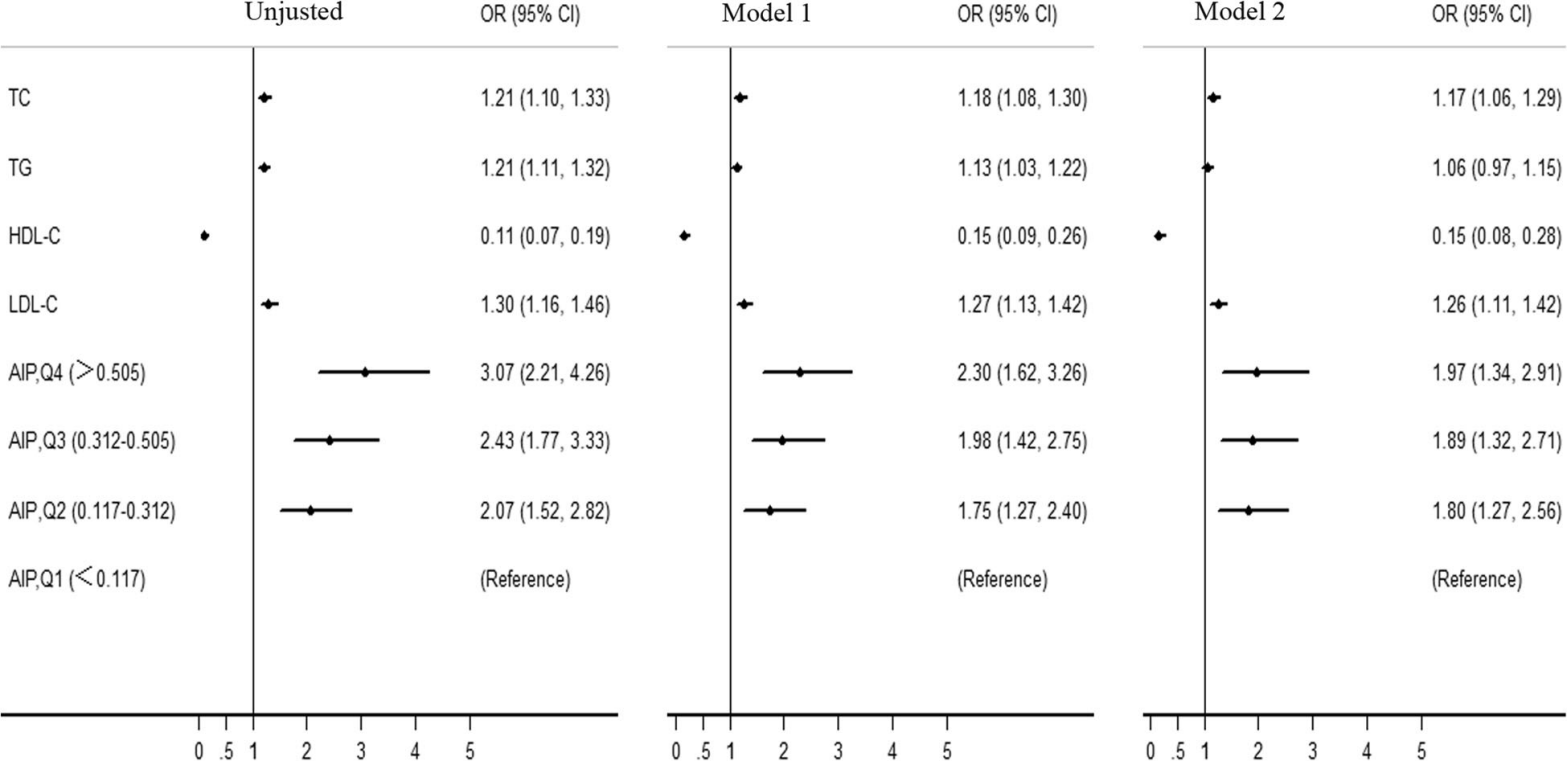
Multivariate logistic regression analysis of the risk of ACS with AIP on quartile scales

Univariate and Multivariate logistic regression analyses were also used to explore the association of AIP with ACS risk (Tables 3 and 4). After adjustment for traditional confounders, we found that AIP remained to be independently associated with the presence of ACS (for ACS, OR = 2.390, 95% CI = 1.855–4.627, *P* < 0.001; for AMI, OR = 3.872, 95% CI = 2.280–6.576, *P* < 0.001; for UAP, OR = 2.151, 95% CI = 1.303–3.549, *P* = 0.003), which was superior to traditional lipid profiles. Moreover, patients with quartile 4 AIP had 1.97-fold risk for ACS (95% CI = 1.27–2.56), compared with those with quartile 1 (Fig. 2). Subgroup analysis stratified by gender was also carried out. Interestingly, the independent association of AIP with ACS risk only existed in male subgroup (Table 4).

**Table 3:** Univariate regression analysis of the association of ACS with variables

Variables	OR	95% CI	*P*
Age	1.043	1.009–1.078	**0.012**
Male	2.579	(1.727–3.851)	**< 0.001**
BMI	1.079	1.049–1.110	**< 0.001**
Smoker	1.974	1.563–2.495	**< 0.001**
Drinker	0.906	0.698–1.176	0.458
EH	1.591	1.240–2.043	**< 0.001**
DM	2.092	1.304–3.355	**0.002**
Dyslipideamia	0.873	0.605–1.261	0.469
PALB	0.008	0.001–0.052	**< 0.001**
TC	1.209	1.101–1.327	**< 0.001**
TG	1.211	1.111–1.321	**< 0.001**
HDL-C	0.111	0.066–0.186	**< 0.001**
LDL-C	1.299	1.157–1.459	**< 0.001**
AIP	4.707	3.184–6.960	**< 0.001**

*ACS* acute coronary syndrome, *BMI* body mass index, *DM* diabetes mellitus, *EH* essential hypertension, *PALB* pre-albumin, *TC* total cholesterol, *TG* triglyceride, *HDL-C* high-density lipoprotein cholesterol, *LDL-C* low-density lipoprotein cholesterol, *AIP* atherogenic index of plasma, *OR* odds ratio, *CI* confidence interval

Bold values indicate statistical significance

**Table 4:** Multivariate logistic regression analysis of the risk of ACS with lipid parameters on a continuous scale

	Lipid parameters	Model 1			Model 2		
		OR	95% CI	*P*	OR	95% CI	*P*
Total	TC	1.183	1.077–1.299	**< 0.001**	1.152	1.048–1.266	**0.003**
	TG	1.125	1.034–1.223	**0.006**	1.078	0.991–1.172	0.079
	HDL-C	0.150	0.088–0.259	**< 0.001**	0.134	0.075–0.241	**< 0.001**
	LDL-C	1.269	1.131–1.424	**< 0.001**	1.046	1.015–1.078	**0.004**
	AIP	3.276	2.159–4.969	**< 0.001**	2.930	1.855–4.627	**< 0.001**
AMI	TC	1.221	1.093–1.365	**< 0.001**	1.162	1.034–1.305	**0.012**
	TG	1.154	1.052–1.267	**0.003**	1.096	0.995–1.208	0.062
	HDL-C	0.124	0.066–0.235	**< 0.001**	0.091	0.044–0.188	**< 0.001**
	LDL-C	1.338	1.166–1.536	**< 0.001**	1.278	1.105–1.479	**0.001**
	AIP	4.284	2.664–6.889	**< 0.001**	3.872	2.280–6.576	**< 0.001**
UAP	TC	1.158	1.043–1.286	**0.006**	1.150	1.033–1.279	**0.010**
	TG	1.087	0.996–1.185	0.060	1.064	0.975–1.161	0.164
	HDL-C	0.181	0.094–0.349	**< 0.001**	0.179	0.091–0.352	**< 0.001**
	LDL-C	1.217	1.074–1.378	**0.002**	1.214	1.069–1.378	**0.003**
	AIP	2.314	1.443–3.711	**< 0.001**	2.151	1.303–3.549	**0.003**
Male	TC	1.173	1.062–1.296	**0.002**	1.142	1.032–1.264	**0.010**
	TG	1.118	1.027–1.218	**0.010**	1.085	0.994–1.184	0.068
	HDL-C	0.195	0.110–0.344	**< 0.001**	0.157	0.085–0.291	**< 0.001**
	LDL-C	1.243	1.100–1.406	**< 0.001**	1.218	1.074–1.381	**0.002**
	AIP	2.996	1.942–4.620	**< 0.001**	2.917	1.817–4.685	**< 0.001**
Female	TC	1.221	1.093–1.365	**< 0.001**	1.362	1.006–1.844	**0.045**
	TG	1.424	0.814–2.492	0.215	1.084	0.692–1.699	0.725
	HDL-C	0.014	0.001–0.135	**< 0.001**	0.024	0.002–0.257	**0.002**
	LDL-C	1.471	1.016–2.131	**< 0.001**	1.218	1.074–1.381	**0.002**
	AIP	8.844	1.605–48.734	**0.012**	4.393	0.621–31.081	0.138

*ACS* acute coronary syndrome, *AMI* acute myocardial infarction, *TC* total cholesterol, *TG* triglyceride, *HDL-C* high-density lipoprotein cholesterol, *LDL-C* low-density lipoprotein cholesterol, *AIP* atherogenic index of plasma, *OR* odds ratio, *CI* confidence interval

Model 1, adjusted for gender, age, smoker, EH and DM; Model 2, adjusted for confounders in model 1 plus BMI; Bold values indicate statistical significance

Additionally, sensitive analysis was performed by calculating the results again when omitting participants prior receiving lipid-lowing drugs. As expected, the results were not obviously changed (data not shown).

## Discussion

To our knowledge, the present study firstly evaluated the relationship between AIP and ACS risk in very young adults (≤35 years old) in a large scale. We found that AIP was independently associated with the presence and severity of ACS in very young patients in a gender-dependent manner, which was superior to traditional lipid profiles.

In past decade, the incidence of young CAD increased greatly [18]**.** Numerous studies have shown significant differences in the risk factors, clinical presentation and angiographic profile between young and older patients with ACS [19]. In the present study, total of 1, 478 participants were enrolled, including 419 non-CAD subjects and 1, 059 ACS patients. In our study, males were more likely to be with ACS. The prevalence of smoker, DM and EH were also significantly higher in very young ACS patients compared with non-CAD subjects.

As a surrogate of the small low-density lipoprotein particle size and needing no extra cost [20], AIP was proposed to be an economic and reliable indicator for CAD clinically. Epidemiological studies showed that the mean AIP level was varied in different populations. According to the report of Northeast China Rural Cardiovascular Health Study, the prevalence of high AIP (> 0.21) was 23.1% in middle-aged subjects in the rural areas of northeast China [21]. However, the AIP level was 0.46 in Turkey population [22] and − 0.1 in staff of an university in Malaysia [9]. Besides the ethnicity and region, diets and physical activity could lead to the difference in AIP in different studies [23–25]. The median level of AIP in whole population was 0.312 in our study, which was higher than that in South China and lower than Turkey population. This discrepancy might be partly interpreted by difference in age, region and ethnicity.

Evidence from epidemiological and clinical analyses showed an independent association between AIP and risk factors for cardiovascular disease. In 2018, Shen SW et al [26] suggested that AIP was linearly correlated with waist circumference and was used as a reference to estimate abdominal obesity. Besides, AIP was independently associated with CAD risk in male and multi-vessel lesson patients after adjustment for risk confounders [27].

Despite promising evidence on AIP as a novel biomarker of CAD, current data were inadequate for its use clinically. Additionally, despite very young ACS accounting for a growing proportion of sudden death in young people and the terrible influence on society and family, there were limited studies investigating the effect of AIP on the risk of ACS in very young patients. Thus, we conducted this large-scale observational study to evaluate the association of AIP with ACS risk in very young participants. Our results suggested that the very young patients with ACS had higher AIP level than non-CAD participants (0.35 ± 0.30 vs 0.21 ± 0.33, *P* < 0.001). Further, we evaluated the association of AIP with the severity of ACS in very young patients. In ACS patients, the AIP gradually increased with the elevated GS score and number of lesion vessels (*P*_for trend_ all< 0.05). Multivariate logistic regression analyses suggested that AIP remained to be independently associated with the presence of ACS and was superior to traditional lipid profiles after adjustment for traditional confounders. In very young adults, AIP level might be a better predictor for ACS, and management and control of AIP level might be an important effect to reduce the ACS risk.

Previous studies suggested that there was a gender dependent difference in AIP and CAD [27]. In a cross-sectional study conducted among 108 Cameroonian postmenopausal women in Cameroon, AIP was found that might not be an independent factor impacting the risk of CVD after adjusting for confounders [28]. However, study conducted in China showed that AIP was a novel and independent predictive indicator for CAD in Chinese postmenopausal women [29]. In the subgroup analysis stratified by gender, we found that AIP was only independently associated with ACS risk in male, which was inconsistent with Wu TT et al [29]*.* However, we must realize that only 105 (7.1%) participants enrolled in the study were female, which might influence the statistic effect. We should interpret it cautiously.

## Limitations

Some limitations of this study should be considered when we interpreted the results. Firstly, the major limitation was the design of this study. The present study was a hospital-based observational study. Although the sample size was large, we could not obtain the cause-result effect, which should be verified by further cohort study. Secondly, we could not ensure the medical history data of every enrolled participant was accurate, also due to the retrospective design. However, the large-scale sample size might reduce the statistic error. Thirdly, 236 (15.97%) very young participants receiving lipid-lowing treatment before enrolled in our study, which might affect the results. So, we carried out the sensitivity analysis by calculating the results again when omitting these participants and the results were not changed significantly, which suggested the results of the present study were robust and credible.

## Conclusions

Despite the limitations, AIP was independently associated with the presence and severity of ACS in very young patients in a gender-dependent manner, which might be superior to traditional lipid profiles.

## Additional file

Additional file 1: **Table S1.** Correlation analysis of AIP with other variables. **Table S2.** Clinical characteristics according to GS quartiles in very young adults with ACS. **Table S3.** Clinical characteristics according to number of lesion vessels in very young adults with ACS. (DOCX 18 kb)

## Abbreviations

ACS: Acute coronary syndrome
AIP: Atherogenic index of plasma
CAD: Coronary artery disease
CAG: Coronary angiography
DM: Diabetes mellitus
EH: Essential hypertension
HDL-C: Highdensity lipoprotein cholesterol
LDL-C: Low-density lipoprotein cholesterol
TC: Total cholesterol
TG: Triglyceride
