# Supplementary material for: Gender-specific associations between atherogenic index of plasma and the presence and severity of acute coronary syndrome in very young adults: a hospital-based observational study
Source: Lipids Health Dis. 2019 Apr 13;18:99. doi: 10.1186/s12944-019-1043-2 (PMC6466804; doi:10.1186/s12944-019-1043-2)
Supplement: Supplementary file 1 — Table S1. Correlation analysis of AIP with other variables. Table S2. Clinical characteristics according to GS quartiles in very young adults with ACS. Table S3. Clinical characteristics according to number of lesion vessels in very young adults with ACS. (DOCX 18 kb) [file 12944_2019_1043_MOESM1_ESM.docx]

**Supplemental table 1. Correlation analysis of AIP with other variables**

|  | Age | BMI | GA | PALB | TC | TG | HDL-C | LDL-C |
| --- | --- | --- | --- | --- | --- | --- | --- | --- |
| r | 0.163 | 0.312 | 0.128 | 0.209 | 0.280 | 0.813 | -0.573 | 0.076 |
| *P* | <0.001 | <0.001 | <0.001 | <0.001 | <0.001 | <0.001 | <0.001 | 0.003 |

BMI, body mass index; GA, glycated albumin; PALB, pre-albumin; TC, total cholesterol; TG, triglyceride; HDL-C, high-density lipoprotein cholesterol; LDL-C, low-density lipoprotein cholesterol; AIP, atherogenic index of plasma.

**Supplemental table 2. Clinical characteristics according to GS quartiles in very young adults with ACS**

| Characteristics | Quartile 1 (≤12) | Quartile 2 (12-32) | Quartile 3 (32-64) | Quartile 4 (>64) | *P_for trend_* |
| --- | --- | --- | --- | --- | --- |
| Clinical Characteristics |  |  |  |  |  |
| Age, years | 33(30-34) | 33(31-34) | 33(31-34) | 33(31-35) | 0.335 |
| Male, n(%) | 240 (94.49) | 293 (93.91) | 215 (93.89) | 257 (97.35) | 0.142 |
| BMI, kg/m^2^ | 27.56±4.10 | 28.22±4.30 | 28.79±4.17 | 28.07±3.93 | 0.094 |
| Smoker, n(%) | 167 (65.75) | 224(71.79) | 163(71.18) | 198(75.00) | **0.033** |
| EH, n(%) | 86(33.86) | 110(35.26) | 102(44.54) | 91(34.47) | 0.447 |
| DM, n(%) | 16(6.30) | 23(7.37) | 33(14.41) | 38(14.39) | **<0.001** |
| Dyslipidaemia, n(%) | 28(11.02) | 26(8.33) | 21(9.17) | 25(9.47) | 0.680 |
| Laboratory parameters |  |  |  |  |  |
| CR, mmol/L | 77.97±14.63 | 76.36±17.31 | 76.76±17.28 | 76.34±15.2 | 0.320 |
| FBG, mmol/L | 5.65±1.44 | 5.91±1.84 | 5.90±1.79 | 6.24±2.38 | **0.001** |
| TC, mmol/L | 4.30±1.14 | 4.55±1.21 | 4.87±1.63 | 4.99±1.66 | **<0.001** |
| TG, mmol/L | 1.81(1.28-2.62) | 1.97(1.28-3.02) | 2.01(1.39-2.83) | 1.98(1.38-3.06) | 0.077 |
| HDL-C, mmol/L | 0.92±0.20 | 0.94±0.23 | 0.89±0.18 | 0.90±0.20 | **0.020** |
| LDL-C, mmol/L | 2.69±1.01 | 2.82±0.94 | 3.17±1.35 | 3.24±1.44 | **<0.001** |
| AIP | 0.32±0.27 | 0.34±0.33 | 0.38±0.27 | 0.38±0.32 | **0.010** |

ACS, acute coronary syndrome; BMI, body mass index; DM, diabetes mellitus; EH, essential hypertension; CR, creatinine; FBG, fasting blood glucose; TC, total cholesterol; TG, triglyceride; HDL-C, high-density lipoprotein cholesterol; LDL-C, low-density lipoprotein cholesterol; AIP, atherogenic index of plasma; GS, Gensini score.

Bold values indicate statistical significance.

**Supplement table 3. Clinical characteristics according to number of lesion vessels in very young adults with ACS**

| Characteristics | One (n=519) | Two (n=244) | Three (n=192) | *P* |
| --- | --- | --- | --- | --- |
| Clinical Characteristics |  |  |  |  |
| Age, years | 33 (30-34) | 33 (31-35) | 33 (31-35) | **<0.001** |
| Male, n(%) | 495 (95.38) | 228 (93.44) | 185 (96.35) | 0.350 |
| BMI, kg/m^2^ | 27.82±4.12 | 28.88±4.15 | 28.53±4.03 | **0.003** |
| Smoker, n(%) | 368 (70.91) | 169 (69.26) | 145 (75.52) | 0.342 |
| EH, n(%) | 168 (32.37) | 115 (47.13) | 81 (42.19) | **0.002** |
| DM, n(%) | 38 (7.32) | 28 (11.48) | 38 (19.79) | **<0.001** |
| Dyslipidaemia, n(%) | 45 (8.67) | 24 (9.84) | 19 (9.90) | 0.560 |
| Laboratory parameters |  |  |  |  |
| TC, mmol/L | 4.43±1.16 | 4.99±1.60 | 5.25±1.84 | **<0.001** |
| TG, mmol/L | 1.88 (1.27-2.78) | 2.00 (1.54-3.06) | 2.16 (1.35-3.50) | **0.001** |
| HDL-C, mmol/L | 0.95±0.22 | 0.89±0.18 | 0.86±0.20 | 0.050 |
| LDL-C, mmol/L | 2.77±0.97 | 3.22±1.31 | 3.44±1.59 | **<0.001** |
| AIP | 0.32±0.30 | 0.39±0.29 | 0.42±0.33 | **<0.001** |

ACS, acute coronary syndrome; BMI, body mass index; DM, diabetes mellitus; EH, essential hypertension; TC, total cholesterol; TG, triglyceride; HDL-C, high-density lipoprotein cholesterol; LDL-C, low-density lipoprotein cholesterol; AIP, atherogenic index of plasma; GS, Gensini score.

Bold values indicate statistical significance.
